# High expression of erythropoietin-producing hepatoma cell line-B2 (EphB2) predicts the efficiency of the Qingyihuaji formula treatment in pancreatic cancer CFPAC-1 cells through the EphrinB1-EphB2 pathway

**DOI:** 10.3892/ol.2014.2134

**Published:** 2014-05-12

**Authors:** YONG-QIANG HUA, ZHEN CHEN, ZHI-QIANG MENG, HAO CHEN, JIAN-GANG SHEN, KUN WANG, WANG PENG, YE-HUA SHEN, LU-MING LIU

**Affiliations:** 1 Department of Integrative Hepatobiliary and Pancreatic Oncology, Fudan University Shanghai Cancer Center, Shanghai 200032, P.R. China; 2 Department of Oncology, Shanghai Medical College, Fudan University, Shanghai 200032, P.R. China; 3 School of Chinese Medicine, University of Hong Kong, Hong Kong, SAR, P.R. China

**Keywords:** erythropoietin-producing hepatoma cell line-B2, pancreatic cancer, Qingyihuaji formula, predictive factor

## Abstract

Our previous study demonstrated that inhibition of erythropoietin-producing hepatoma cell line-B2 (EphB2) expression resulted in the promotion of cancer growth, with EphB2 acting as a tumor suppressor in pancreatic cancer. Qingyihuaji formula (QYHJ), a traditional Chinese medicine, acts as an independent protective factor for pancreatic cancer patient survival and different patients have shown various responses to QYHJ treatment. In the current study, the different effects on tumor growth inhibition following QYHJ treatment in cells with different levels of EphB2 expression were investigated to reveal the mechanism. A subcutaneously transplanted tumor model using cancer cells with different levels of EphB2 expression were established *in vivo* and received a four-week QYHJ intervention. Tumor weight inhibitory rate and tumor volume deflation were evaluated. The cell cycle and apoptosis were analyzed by flow cytometry, and reverse transcription polymerase chain reaction and western blot analysis were used to assess mRNA and protein levels. The results showed that the tumor weight inhibitory rate was 31.40, 31.33 and 18.36% in CFPAC-1, CFPAC-1 control RNAi and CFPAC-1 EphB2 RNAi cells following QYHJ treatment, respectively. A statistically significant difference was identified in CFPAC-1 (P<0.05) and CFPAC-1 control RNAi (P<0.01) cells. In addition, a statistically significant increase was identified in the G0/G1 phase population (P<0.05) and a statistically significant decrease was identified in the S phase population (P<0.05) in CFPAC-1 and CFPAC-1 control RNAi cells; however, no significant difference was identified in the CFPAC-1 EphB2 RNAi cells following QYHJ treatment. QYHJ upregulated the mRNA and protein level of Eph receptor-interacting B1 (EphrinB1) in the cells that were expressing different levels of EphB2, however, QYHJ did not regulate EphB2 expression. In CFPAC-1 and CFPAC-1 control RNAi cells, the QYHJ treatment resulted in a statistically significant decrease in cyclin-dependent kinase 6 (CDK6) mRNA (P<0.05) and protein (P<0.05) levels. The high expression of EphB2 predicted the superior response rate to the QYHJ treatment through a mechanism of inhibiting the cell cycle by an EphrinB1-EphB2-induced CDK6 decrease in CFPAC-1 cells. Therefore, EphB2 acts as a predictive factor for QYHJ treatment in pancreatic cancer CFPAC-1 cells.

## Introduction

Pancreatic cancer is a highly lethal disease and tumors are often late stage at diagnosis, at which point there are few effective treatment options. In 2012, pancreatic cancer resulted in ~85.13% of cancer-related mortality within the United States, the five-year survival rate was ≤5% and the median survival was less than six months (1). Notable improvements have been made in the five-year survival rates for a number of cancers over the past 30 years, with the exception of pancreatic cancer (1,2). Therefore, it is imperative to identify novel treatment strategies to prolong patient survival time for pancreatic cancer. Qingyihuaji formula (QYHJ) has been used in pancreatic cancer treatment for a number of years at the Fudan University Cancer Center (Shanghai, China). Our previous retrospective studies have shown that QYHJ treatment prolongs the survival time of pancreatic cancer patients, with multivariate analysis demonstrating that QYHJ acted as an independent protective factor for pancreatic cancer with liver metastases (3–5). However, QYHJ appeared to prolong the survival time for one subgroup of patients, however, was ineffective for a different group of patients. Our previous study showed that different pancreatic cancer cell lines with different levels of erythropoietin-producing hepatoma cell line-B2 (EphB2) expression exhibited different responses to QYHJ treatment (6). It has previously been confirmed that EphB2 is a prognostic factor for several types of cancer and acts preferentially as a tumor suppressor in various cancers (7–15). Furthermore, our previous study revealed that inhibition of EphB2 expression in pancreatic cancer CFPAC-1 cells resulted in the promotion of cancer growth by stimulating cell proliferation and decreasing apoptosis. Therefore, EphB2 acts as a tumor suppressor in pancreatic cancer (16). Traditional Chinese medicine (TCM) is regarded as a multi-target therapy, which is similar to target therapy in modern medicine (17,18). Previous studies revealed that not all patients benefit from target therapies as patients may have their own effective population according to their distinctive predictors. Thus, the aim of the present study was to investigate the correlation between the different levels of EphB2 expression and the response to QYHJ treatment. In addition, to elucidate whether EphB2 acts as a predictive factor for QYHJ treatment.

## Materials and methods

### Cell cultures

In 1990, the human pancreatic cancer CFPAC-1 cell line was established from a patient with cystic fibrosis by Schoumacher *et al* (19). CFPAC-1 EphB2 RNAi and CFPAC-1 control RNAi cells were transfected by lentivirus-based RNAi to inhibit EphB2 expression, and served as a control RNAi in our previous study (16). Cells were cultured in RPMI-1640 medium (Gibco-BRL, Carlsbad, CA, USA) with 10% heat-inactivated fetal bovine serum (Hyclone, Logan, UT, USA) under a 5% CO_2_ atmosphere at 37°C. The medium was changed at 24 h intervals when the culture had almost reached confluence.

### Drug preparation and intervention

QYHJ is composed of *Hedyotidis herba*, *Amorphophallus konjac*, *Herba scutelleriae barbatae*, coix seed, akebia stem, *Gynostemma pentaphyllum* and java amomum fruit. The herb powder was produced by Jiangyin Tianjiang Pharmaceutical Co., Ltd., (Jiangyin, China). QYHJ was prepared by dissolving the herb powder into distilled water to the required concentration. The daily dosage of QYHJ for the nude mice was calculated according to the following human-mouse transfer formula: D_b_ = D_a_ × (R_b_/R_a_) × 2/3 (W_b_/W_a_) where D, R, and W represent dosage, weight coefficient and body weight, respectively, and a and b represent human and mouse, respectively. The QYHJ group received a total of 200 μl liquid QYHJ twice a day by oral gavage as well as a 36 g/kg daily dosage, the gemcitabine group received an intraperitoneal injection of 120 mg/kg gemcitabine on days one, eight and 15 and the control group received an oral gavage of a total of 200 μl normal saline twice a day. All of the animal studies were reviewed and approved by the Animal Care and Use Committee of Fudan University (Shanghai, China) and were in accordance with the guidelines of the Department of Health and Human Services.

### Assessment of tumorigenicity in vivo

In total, 1×10^6^ CFPAC-1, CFPAC-1 control RNAi and CFPAC-1 EphB2 RNAi cells (200 μl) with different levels of EphB2 expression were injected subcutaneously into the right flank of eight-week-old female BALB/c nude mice. Tumor volume was measured twice per week and calculated using the following formula: Tumor volume = 0.52 × A × B^2^, where A is the length (long diameter) and B is the width (short diameter) of the tumor. Following four weeks of intervention with drugs, the mice were sacrificed, the tumors were dissected and the tumor weight was measured. The tumor weight inhibitory rate was calculated according to the following formula: Tumor weight inhibitory rate = 100 × (tumor weight of control group - tumor weight of QYHJ group) / tumor weight of control group.

### Cell cycle and apoptosis analyses

The tumors were dissected, ground, centrifuged and washed with phosphate-buffered saline (PBS). Next, 1×10^6^ cells were fixed in 1 ml ethanol at 4°C for 1 h. Following centrifugation at 1,500 × g for 10 min (L-550, Changsha Xiangyi Centrifuge Instrument Co., Ltd., Changsha, China) and washing with PBS, the cells were resuspended in 250 μl PBS containing 12.5 μg RNase and incubated for 30 min at 37°C. Cellular DNA was stained with 250 μl propidium iodide (PI) for 30 min at room temperature in the dark. The stained cells were analyzed by flow cytometry (FCM) for cell cycle analysis, and 1×10^6^ cells were centrifuged at 400 × g for 10 min and resuspended in 250 μl PBS; these were incubated for 10 min at 37°C with 1 μg/ml Heochst 33342. Following centrifugation and washing with PBS, the cells were resuspended in 1 ml PI and incubated for an additional 15 min at room temperature in the dark. The stained cells were analyzed by FCM to assess the cell apoptosis.

### Reverse transcription-polymerase chain reaction analysis (RT-PCR)

Total RNA from the tumor tissue was extracted using TRIzol (Invitrogen Life Technologies, Carlsbad, CA, USA), according to the manufacturer’s instructions. RT was performed using the RevertAid H Minus First Strand cDNA Synthesis kit (Fermentas, Waltham, MA, USA), according to the manufacturer’s instructions. The primer sequences used for the targeted genes were as follows: Sense, 5′-TGTAAACAAACCCAGATGCAGGA-3′ and antisense, 5′-CAGGTACATATCACGCGCACAG-3′ for EphB2; sense, 5′-CGCCGTTGGCCAAGAACCTGG-3′ and antisense, 5′-CAGCTTGTCTCCAATCTTCGG-3′ for Eph receptor-interacting B1 (EphrinB1); sense, 5′-GAAGATCGTCGCCACCTG-3′ and antisense, 5′-GACCTCCTCCTCGCACTTCT-3′ for cyclin D1; sense, 5′-CAGTACGAATGCGTGGCG-3′ and antisense, 5′-CTCCTCGCCGGTCTGCAC-3′ for cyclin-dependent kinase 6 (CDK6); sense, 5′-TTGCTTTACGTGGCCTGTTTC-3′ and antisense, 5′-GAAGACCCTGAAGGACAGCCAT-3′ for Bcl-2; and sense, 5′-GGGAGCCAAAAGGGTCATCATCTC-3′ and antisense, 5′-CCATGCCAGTGAGCTTCCCGTTC-3′ for GAPDH. PCR was performed with 30 cycles of 30 sec at 94°C, 30 sec at 58°C and 45 sec at 72°C. The PCR products were electrophoresed and the bands were visualized under ultraviolet radiation following staining with ethidium bromide. The bands were determined and semi-quantified using Labworks 4.6 software (UVP Products, Upland, CA, USA).

### Western blot analysis

Antibodies against EphB2 and EphrinB1 were obtained from Santa Cruz Biotechnology Inc. (Santa Cruz, CA, USA), and antibodies against cyclin D1, CDK6 and Bcl-2 were obtained from Cell Signaling Technology, Inc. (Beverly, MA, USA). Proteins were extracted from the tumor tissue and the concentration was determined via a bicinchoninic acid assay kit (Pierce Biotechnology, Inc., Rockford, IL, USA). In total, 50 μg protein from each sample was electrophoresed at 180 V for 1.5 h in Tris-glycine running buffer. The proteins were transferred onto a nitrocellulose membrane at room temperature overnight and incubated with EphB2, EphrinB1, cyclin D1, CDK6 or Bcl-2 primary antibodies for 2 h, followed by washing with the second antibody for 1 h. Goat anti-β-actin polyclonal antibody served as an internal control and rabbit anti-goat secondary antibody was subsequently used. The protein expression was detected using an Enhanced Chemiluminescence Plus kit (Amersham Pharmacia Biotech, Amersham, UK), exposure to X-ray film or under ultraviolet radiation. The bands were semi-quantified using Labworks 4.6 software.

### Statistical analysis

Data are presented as the mean ± standard error. Statistical analyses were performed by one-way analysis of variance, followed by the Student-Newman-Keuls test for multiple comparisons to compare the results of the *in vivo* experiments, FCM, and the mRNA and protein levels. P<0.05 was considered to indicate a statistically significant difference.

## Results

### High expression of EphB2 indicates advantageous tumor growth suppression with QYHJ treatment in CFPAC-1 cells

**Figure 1:**
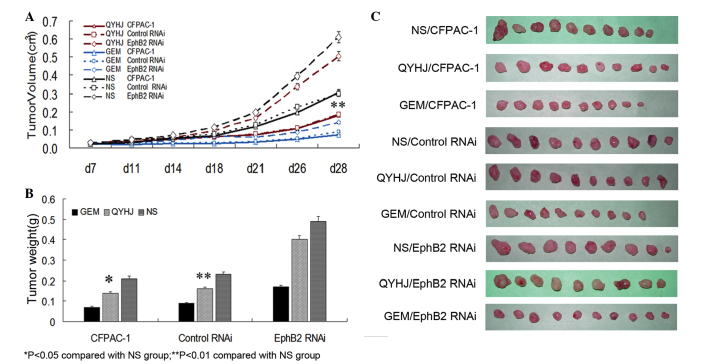
High expression of EphB2 indicates advantageous tumor growth suppression of QYHJ treatment in CFPAC-1 cells. (A) No significant difference was identified in tumor volume following QYHJ treatment in CFPAC-1 EphB2 RNAi cells, however, a significant inhibition was obtained in CFPAC-1 (P<0.05) and CFPAC-1 control RNAi (P<0.05) cells following four weeks of intervention. (B) A statistically significant decrease was identified in tumor weight in CFPAC-1 (P<0.05) and CFPAC-1 Contol RNAi (P<0.01) cells following QYHJ treatment, however, no difference was obtained in CFPAC-1 EphB2 RNAi cells. (C) Tumor weight of nude mice transplanted subcutaneously with CFAPC-1 cells with different levels of EphB2 expression. Each group included nine to 10 nude mice. EphB2, erythropoietin-producing hepatoma cell line-B2; QYHJ, Qingyihuaji formula. ^*^P<0.05 compared with the NS group. **P<0.01 compared with the NS group. NS, normal saline.

CFPAC-1, CFPAC-1 control RNAi and CFPAC-1 EphB2 RNAi cells with different levels of EphB2 expression were injected subcutaneously into female BALB/c nude mice to establish a tumor-bearing mouse model. The mice were divided into QYHJ, gemcitabine and control groups. The QYHJ group received a total of 200 μl liquid QYHJ twice a day by oral gavage, the gemcitabine group received an intraperitoneal injection of 120 mg/kg gemcitabine on days one, eight and 15, and the control group received an oral gavage of a total of 200 μl normal saline twice a day. Tumor volume was measured twice a week for four weeks, and following four weeks of intervention, the tumor weight was measured and the tumor weight inhibitory rate was calculated. The results showed that there was no significant difference in tumor volume between the QYHJ and control groups following 28 days of measuring in the CFPAC-1 EphB2 RNAi cells, however, a significant inhibition was observed in the CFPAC-1 and CFPAC-1 control RNAi cells (P<0.05; Fig. 1A and C). In the QYHJ group, tumor weight was 0.14±0.04, 0.16±0.04 and 0.40±0.10 g for the CFPAC-1, CFPAC-1 control RNAi and CFPAC-1 EphB2 RNAi cells, respectively. In addition, the tumor weight inhibitory rate was 31.40 and 31.33% for CFPAC-1 and CFPAC-1 control RNAi cells, respectively, with a statistically significant decrease in the corresponding QYHJ group compared with the control group (P<0.05, P<0.01); the tumor weight inhibitory rate was only 18.36% in the CFPAC-1 EphB2 RNAi cells, with no statistically significant difference identified between the QYHJ and control groups (Fig. 1B and C). Different levels of EphB2 expression reflected the different responses to the QYHJ treatment. The cells that were expressing higher levels of EphB2 exhibited more effective tumor growth inhibition as a result of the QYHJ treatment, therefore, EphB2 may function as an effective predictive factor for QYHJ treatment.

### QYHJ suppresses tumor growth by retarding the cell cycle process, but not cell apoptosis in CFPAC-1 cells

**Figure 2:**
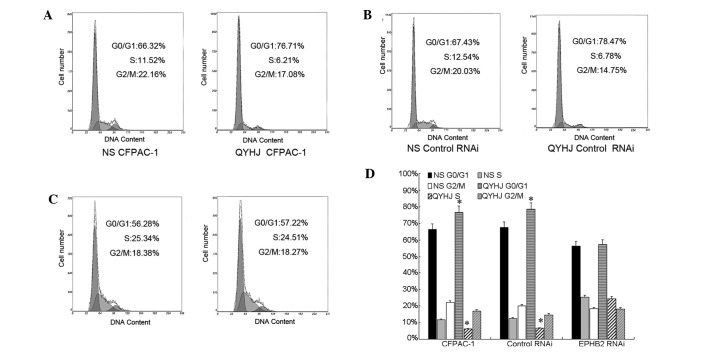
QYHJ suppresses tumor growth through inhibiting the cell cycle process. (A–C) Cell cycle was analyzed in CFPAC-1,CFPAC-1 control RNAi and CFPAC-1 EphB2 RNAi cells by flow cytometry following QYHJ treatment. (D) QYHJ treatment blocked the cell cycle in the G0/G1 phase and reduced the S phase proportion in CFPAC-1 and CFPAC-1 control RNAi cells. A statistically significant increase was identified in the G0/G1 phase population (P<0.05) and a decrease was identified in the S phase population in CFPAC-1 and CFPAC-1 control RNAi cells, however, no difference was identified in the CFPAC-1 EphB2 RNAi cells following QYHJ treatment (P<0.05). EphB2, erythropoietin-producing hepatoma cell line-B2; QYHJ, Qingyihuaji formula. ^*^P<0.05 compared with the NS group.

**Figure 3:**
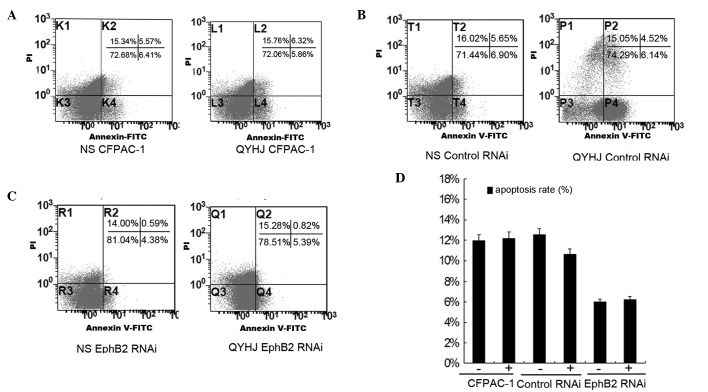
Cell apoptosis is not involved in the inhibition of tumor growth following QYHJ treatment. (A–C) Cell apoptosis was analyzed by flow cytometry following QYHJ treatment. (D) No statistically significant increase was identified in the apoptosis rate following QYHJ treatment in CFPAC-1, CFPAC-1 control RNAi and CFPAC-1 EphB2 RNAi cells. EphB2, erythropoietin-producing hepatoma cell line-B2; QYHJ, Qingyihuaji formula; FITC, fluorescein isothiocyanate.

Our previous study showed that the overexpression of EphB2 suppressed tumor growth by inhibiting the cell cycle process and inducing cell apoptosis in CFPAC-1 cells (16). Accordingly, in the present study, the cell cycle and apoptosis were analyzed by FCM to reveal the mechanisms of the different responses to QYHJ treatment in CFPAC-1 cells, which were expressing different levels of EphB2. In the CFPAC-1 EphB2 RNAi cells, the proportion of cells in the G0/G1 phase was 25.34 and 24.51% and in the S phase was 56.28 and 57.22% for the QYHJ and control groups, respectively (Fig. 2C and D). No significant change was identified in the cell cycle distribution following QYHJ treatment, however, QYHJ treatment blocked the cell cycle in the G0/G1 phase and reduced the proportion of cells in the S phase in CFPAC-1 and CFPAC-1 control RNAi cells. In the CFPAC-1 and CFPAC-1 control RNAi cells, the proportion of cells in the G0/G1 phase was 76.71 and 66.32% in the QYHJ group, respectively, and 78.47 and 67.43% in the control group, respectively. The QYHJ treatment resulted in a statistically significant increase in the G0/G1 phase population in CFPAC-1 and CFPAC-1 control RNAi cells (P<0.05; Fig. 2A,B and D). An ~50% decrease in the S phase proportion was observed following QYHJ treatment in the CFPAC-1 and CFPAC-1 control RNAi cells; the proportion was decreased from 11.52 to 6.21% in the CFPAC-1 cells and from 12.54 to 6.78% in the CFPAC-1 control RNAi cells. In addition, a statistically significant decrease was identified in the S phase population in the CFPAC-1 and CFPAC-1 control RNAi cells following QYHJ treatment (P<0.05; Fig. 2A,B and D). The apoptosis rate was 11.98, 12.55 and 4.97% for the control group in the CFPAC-1, CFPAC-1 control RNAi and CFAC-1 EphB2 RNAi cells, respectively, and was 12.18, 10.68 and 6.21% following QYHJ treatment in the corresponding cell lines (Fig. 3A–C). QYHJ treatment did not result in a statistically significant increase in the proportion of dead cells in CFPAC-1 cells that were expressing different levels of EphB2 (Fig. 3D).

### High expression of EphB2 predicts the superior response to QYHJ treatment via the EphrinB1-EphB2-CDK6 pathway in CFPAC-1 cells

**Figure 4:**
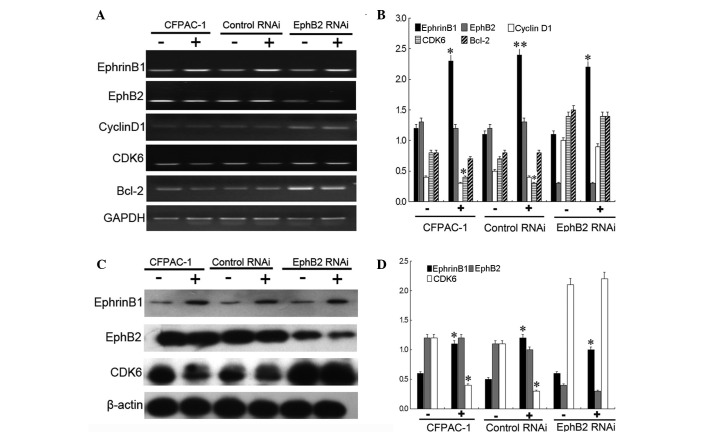
High expression of EphB2 predicts a superior response to QYHJ treatment through the EphrinB1-EphB2-CDK6 pathway in CFPAC-1 cells. QYHJ resulted in an unclear change of EphB2 (A and B) mRNA and (C and D) protein level, however, a statistically significant increase was identified in the EphrinB1 (A and B) mRNA (P<0.05, P<0.01 and P<0.05) and (C and D) protein (P<0.05, P<0.05 and P<0.05) level following QYHJ treatment in CFPAC-1, CFPAC-1 control RNAi cells and CFPAC-1 EphB2 RNAi cells. QYHJ also resulted in a statistically significant decrease in the CDK6 (A and B) mRNA (P<0.05) and (C and D) protein (P<0.05) level in CFPAC-1 and CFPAC-1 control RNAi cells (P<0.05), however, no change was identified in CFPAC-1 EphB2 RNAi cells. EphB2, erythropoietin-producing hepatoma cell line-B2; QYHJ, Qingyihuaji formula; CDK6, cyclin-dependent kinase 6; EphrinB1, Eph receptor-interacting B1. ^*^P<0.05 and ^**^P<0.01 compared with the NS group.

Our previous study found that EphB2 induced the G0/G1 phase by blocking the downregulation of cyclin D1 and CDK6 in the CFPAC-1 cells (16). Previous studies have revealed that EphB2 emanates the intracellular signal by binding the transmembrane EphrinB1 ligands to form the Eph-EphrinB1 complex, which is required for EphB2 kinase activity transmission in the receptor-expressing cells (20,21). In addition, decreased cell growth has been observed in EphB2-expressing tumor cells in the presence of the EphrinB1/Fc ligand (22). An additional experiment was performed in the present study to verify the mechanism of the superior response to QYHJ treatment in cells, which were expressing higher levels of EphB2. The results showed that the mRNA and protein levels of EphB2 changed indistinctly, following QYHJ treatment, in the CFPAC-1, CFPAC-1 control RNAi and CFPAC-1 EphB2 RNAi cells of the subcutaneous tumor. QYHJ significantly increased the mRNA and protein level of EphrinB1 in these three cell lines and a statistically significant increase was identified in the EphrinB1 mRNA (P<0.05, P<0.01 and P<0.05) and protein (P<0.05, P<0.05 and P<0.05) level between the corresponding QYHJ and control groups. Furthermore, the QYHJ treatment resulted in the statistically significant downregulation of CDK6 mRNA (P<0.05) and protein (P<0.05) levels in the CFPAC-1 and CFPAC-1 control RNAi cells, however, did not affect cyclin D1 expression (Fig. 4). Cell cycle-related CDK6 and cyclin D1 did not show a statistically significant change following QYHJ treatment in the CFPAC-1 EphB2 RNAi cells (Fig. 4). In addition, QYHJ treatment did not result in a statistically significant change in cell apoptosis-related Bcl-2 expression in cells that were expressing different levels of EphB2 (Fig. 4A and B). Consequently, the mechanism of the different cancer growth inhibition responses following QYHJ treatment did not necessarily arise as a result of the upregulation of EphB2, rather, it is the critical upregulation of EphrinB1 that stimulates EphB2-expressing cells to inhibit cancer cell growth by downregulating CDK6 expression.

## Discussion

Pancreatic cancer is one of the most malignant types of cancer worldwide. In 2012, pancreatic cancer resulted in ~85.13% of cancer-related mortality in the USA. In addition, the five-year survival rate was ≤5% and the median survival rate was less than six months (1,2). Only 20% of patients are diagnosed at early stage and undergo surgical treatment (23). Currently, chemotherapy or radiation are the best treatment options for >80% of advanced-stage patients, however, a number of these cases are found to be highly resistant to these treatments (24,25). Despite the rapid advancement over the last decade in cancer therapy, pancreatic cancer patients benefit the least with regard to treatment products and survival rate due to the high degree of malignancy, and susceptibility to chemoradiation resistance. Therefore, it is imperative to identify the mechanism of the treatment resistance, which results in a poor prognosis, and to identify novel treatment strategies to prolong patient survival time.

Ephrin receptors are one of the largest subfamilies of receptor tyrosine kinases (RTKs). EphB2 is one of the two subgroups, which preferentially binds transmembrane EphrinB ligands (26–28). Previously, it was verified that EphB2 is a prognostic factor for several types of cancer; it has been associated with histological grade, stage, and overall and disease-free survival (7–12). Numerous studies have demonstrated that EphB2 preferentially acts as a tumor suppressor in various cancers, including colorectal (CRC) (13), gastric (15) and prostate (14) cancer, which is unlike other RTKs that are generally regarded as oncogenes. In CRC, the loss of EphB2 expression is observed in >50% patients, its downregulation accelerates the progression of CRC (21) and is associated with a poor prognosis (9,11,29). A complete loss of EphB2 expression is observed in 52.5% of gastric cancer and 82% of nodal metastases patients. Therefore, loss of EphB2 expression is significantly associated with advanced T stage, nodal metastasis, advanced disease stage and poor histological differentiation. The frequent deletion and decreased expression of EphB2 indicates that it may be a negative biomarker for gastric cancer and a potential predictor of the final outcome (15). In addition, EphB2 has been identified as a tumor suppressor gene in prostate cancer (14). Despite the clear link between EphB2 and a number of cancers, little is known concerning the correlation between EphB2 and pancreatic cancer prognosis. Our previous study was designed to investigate this correlation by eliminating EphB2 expression using lentivirus-based RNAi to observe the biological characteristic changes in pancreatic cancer CFPAC-1 cells. The results demonstrated that silencing EphB2 promoted cancer growth by stimulating cell proliferation through a mechanism of G1/S phase breakthrough, which was dependent on a cyclin D1/CDK6 cell cycle regulating signal. Similarly, EphB2 inhibition also reduced the apoptosis of CFPAC-1 cells by increasing Bcl-2 expression, with EphB2 acting as a tumor suppressor in the cell proliferation and apoptosis in pancreatic cancer (16).

For thousands of years, TCM has been widely used for cancer treatment in China (30). Integrative TCM and Western medicine for cancer treatment has been broadly approved by governments and patients, including for pancreatic cancer. The authors of the present study have accumulated a wealth of experience in pancreatic cancer integrative therapy and obtained certain promising results. A total of 164 pancreatic cancer patients with liver metastases that were treated with chemotherapy, radiation therapy and/or QYHJ were analyzed. The results demonstrated an overall median survival time of 4.7 months and a one-year survival time of 14%, with clinical outcomes more effective than those identified by other studies (31). Multivariate analysis showed that chemotherapy and QYHJ were protective factors (3). Notably, certain patients experienced long survival times when treated with QYHJ, however, disease continued to progress in a number of patients even though QYHJ treatment had been received in clinical practice. These differences in patient reaction to QYHJ treatment require investigation; there may be effective and ineffective populations for QYHJ treatment, therefore, a method to distinguish them must be identified. TCM is regarded as a multi-target therapy through immune alteration, tumor microenvironment transforming, oncogenes or tumor suppressor gene regulation, and is comparable to target therapy in modern medicine (17,18). Previous studies have identified that not all patients benefit from target therapies since patients have their own effective population according to their distinctive predictors. Several types of treatment response predictors have been identified for target therapy, and gene expression predictors have been regarded as the most appropriate and valuable as they organically link the molecular biology and pharmacology (32–34). Aberrant activation or mutations in RTKs are responsible for tumor progression and development, and a number of RTKs have been validated as prognostic factors and therapeutic targets in human cancers, which are caused by activated RTKs (35–37). In addition, specific RTKs have been verified as able to show a predictive value for target therapy (38,39). Epidermal growth factor receptor mutations appear to identify distinct subsets of patients with an increased response to gefitinib in non-small cell lung carcinoma (38). In CRC, a KRAS mutation has previously been associated with resistance to cetuximab and a poorer survival in metastatic CRC patients that were treated with cetuximab (39). In pancreatic cancer, histone levels are a survival predictor for patients who have received adjuvant fluorouracil; furthermore, histone modification patterns predicted the prognosis and the treatment response (33).

EphB2 functions as a positive prognostic factor and tumor suppressor in pancreatic cancer growth and our previous studies identified that different pancreatic cancer cell lines appear to exhibit different responses to QYHJ, as cells expressed different levels of EphB2 (6). By acting as a positive prognostic factor in pancreatic cancer, or a distinctive predictive factor for QYHJ treatment, partially reveals the mechanism of the different responses to QYHJ treatment in cells that express different levels of EphB2. Subsequently, a series of experiments were performed in the present study to verify this hypothesis. The results showed that tumor weight was 0.14±0.04, 0.16±0.04 and 0.40±0.10 g for CFPAC-1, CFPAC-1 control RNAi and CFPAC-1 EphB2 RNAi cells, respectively, and the tumor weight inhibitory rate was 31.40, 31.33 and 18.36%, respectively. A statistically significant decrease was identified in tumor weight following the QYHJ intervention in CFPAC-1 (P<0.05) and CFPAC-1 control RNAi (P<0.01) cells. However, a statistically significant difference was predicted to appear in CFPAC-1 EphB2 RNAi cells following the QYHJ treatment. Previously, a high expression of EphB2 was found to indicate advantageous tumor growth suppression with QYHJ treatment in CFPAC-1 cells. This is comparable to the clinical results, which were identified in the present study, that QYHJ acts as a protective factor and prolongs survival time for certain patients, however, not all patients. Cell cycle analyses showed that QYHJ treatment did not change the cell cycle distribution in CFPAC-1 EphB2 RNAi cells, however, a statistically significant increase in the G0/G1 phase (P<0.05), and a significant decrease in the S phase (P<0.05) populations was identified in CFPAC-1 and CFPAC-1 control RNAi cells following QYHJ treatment. QYHJ treatment did not result in a statistically significant increase in the proportion of dead cells in CFPAC-1 cell lines that were expressing different levels of EphB2. Accordingly, a higher expression of EphB2 acted as a positive predict factor for QYHJ treatment, with the exception of being a prognostic factor in pancreatic cancer; an additional experiment was performed to explore this mechanism. Previous studies revealed that the overexpression or loss of EphB2 influenced the cell cycle and apoptosis (12,40,41) and that EphB2 emanated intracellular signals by binding EphrinB1 ligands to form the EphB2-EphrinB1 complex (20,21,27). The results of the current study showed that the mRNA and protein level of EphB2 changed indistinctly following QYHJ treatment. In addition, QYHJ significantly increased the mRNA and protein level of EphrinB1 in CFPAC-1 cell lines that were expressing different levels of EphB2. The QYHJ treatment resulted in a statistically significant decrease in the S phase population, which was associated with a decrease in the CDK6 mRNA (P<0.05) and protein (P<0.05) levels in CFPAC-1 and CFPAC-1 control RNAi cells. No evident change was observed in the CFPAC-1 EphB2 RNAi cells. Furthermore, QYHJ treatment did not result in a statistically significant change in Bcl-2 expression in cells that were expressing different levels of EphB2. The mechanism of the higher expression of EphB2 acting as a predictive factor for QYHJ treatment arose as a result of the upregulation of EphrinB1, which stimulated the EphB2-expressing cells to inhibit cancer cell growth by downregulating the CDK6 expression.

In conclusion, a high expression of EphB2 predicts a superior response to QYHJ treatment through a mechanism that is dependent on inhibiting the cell cycle via an EphrinB1-EphB2-induced CDK6 decrease in CFPAC-1 cells. Therefore, EphB2 may act as a predictive factor for QYHJ treatment in pancreatic cancer CFPAC-1 cells.
